# A New Statistical Approach to Characterize Chemical-Elicited Behavioral Effects in High-Throughput Studies Using Zebrafish

**DOI:** 10.1371/journal.pone.0169408

**Published:** 2017-01-18

**Authors:** Guozhu Zhang, Lisa Truong, Robert L. Tanguay, David M. Reif

**Affiliations:** 1 Bioinformatics Research Center, North Carolina State University, Raleigh, North Carolina, United States of America; 2 Department of Environmental and Molecular Toxicology, Sinnhuber Aquatic Research Laboratory, Oregon State University, Corvallis, Oregon, United States of America; 3 Department of Biological Sciences, Center for Human Health and the Environment, North Carolina State University, Raleigh, North Carolina, United States of America; University Zürich, SWITZERLAND

## Abstract

Zebrafish have become an important alternative model for characterizing chemical bioactivity, partly due to the efficiency at which systematic, high-dimensional data can be generated. However, these new data present analytical challenges associated with scale and diversity. We developed a novel, robust statistical approach to characterize chemical-elicited effects in behavioral data from high-throughput screening (HTS) of all 1,060 Toxicity Forecaster (ToxCast™) chemicals across 5 concentrations at 120 hours post-fertilization (hpf). Taking advantage of the immense scale of data for a global view, we show that this new approach reduces bias introduced by extreme values yet allows for diverse response patterns that confound the application of traditional statistics. We have also shown that, as a summary measure of response for local tests of chemical-associated behavioral effects, it achieves a significant reduction in coefficient of variation compared to many traditional statistical modeling methods. This effective increase in signal-to-noise ratio augments statistical power and is observed across experimental periods (light/dark conditions) that display varied distributional response patterns. Finally, we integrated results with data from concomitant developmental endpoint measurements to show that appropriate statistical handling of HTS behavioral data can add important biological context that informs mechanistic hypotheses.

## Introduction

A major focus of toxicological research is to develop high-throughput screening (HTS) assays to keep pace with the ever-increasing number of chemicals in commerce while retaining toxicity information, reducing the cost, and the use of animals [1]. HTS in vitro assays, such as Toxicity Forecaster (ToxCast) and Toxicology Testing in the 21^st^ Century (Tox21), were implemented to speed up the pace of chemical testing [2,3]. However, these target-specific technologies do not assay the systems-level bioactivity of chemicals. Thus, new strategies are needed to characterize the hazardous profiles of chemicals and provide complementary, systematic data in order to build computational models to advance toxicological research.

Zebrafish (*Danio rerio*) have become an important model organism for drug discovery and toxicological research, including developmental toxicity and neurotoxicity, due to many great benefits, such as genetic homology to humans, small size, and cost-effectiveness [4–6]. Behavioral profiling in zebrafish has elucidated diverse mechanisms of action [7,8]. There are various behavioral tests that can be performed on developing zebrafish, however, these complex phenotypes introduce many analytical challenges [9,10]. Recent reviews have evaluated currently available statistical methods, including traditional methods such as Student’s T test and analysis of variance (ANOVA), as well as more advanced methods such as behavioral barcoding, multivariate analysis of variance (MANOVA), and pattern matching methods, in addressing different behavioral tests of developing zebrafish [7,8,10,11]. Statistical modeling procedures then aggregate the movement index by a measure of centrality (most often the mean) and connects them across experimental time periods. Modeling the mean movement fails to account for the variability observed across samples that can arise from population genetic diversity, measurement error, or other environmental factors. Moreover, typical behavioral studies are conducted to address a targeted hypothesis, where the scale of resulting data may not be sufficient to robustly characterize outliers. This is especially true when identifying outliers in the negative direction that tend to be closer to the center of mass of right-skewed behavior and thus more difficult to separate, which potentially introduces statistical bias by reducing mean movement.

In this study, we present HTS behavior data from 120hpf zebrafish statically exposed to 1,060 unique ToxCast Phase-I and Phase-II chemicals. We developed a new statistical pipeline to characterize zebrafish behavioral profiles. Our approach is nonparametric, automatically removes outliers at both directions, accounts for inter-individual variability, and significantly reduces the coefficient of variation. Taking advantage of this big data set, we were able to provide diverse diagnostic and verification techniques to prove the concept that our statistical pipeline is robust to the unusual response distributions common in behavioral data and beneficial over existing methods. More importantly, this computational framework can be implemented to any scale of behavioral study in general. Finally, the zebrafish behavioral profiles were compared against concomitantly measured endpoints and mapped against external animal toxicity data to inform diverse mode of action hypotheses.

## Materials and Methods

### Chemicals

The chemical library contains 1,078 (1,060 unique) EPA ToxCast Phase-I and Phase-II chemicals and were provided by the US Environmental Protection Agency. To assess reproducibility, there were 9 chemicals each run in triplicate (as blinded, independent samples). All chemicals were provided in 96 well plates at 20 mM in 100% dimethyl sulfoxide (DMSO). Chemical preparations were conducted according to Truong et al. [12]. Briefly, 8 chemicals were diluted on two plates with the first dilution plate made at 10 mM in 100% DMSO, and underwent 5, 10-fold serial dilution. A 1:15 dilution of plate 1 made up of plate 2, which consisted of 6.4% DMSO. The dilution plates were stored at -20C until time for exposure.

### Experimental design

**Fig 1:**
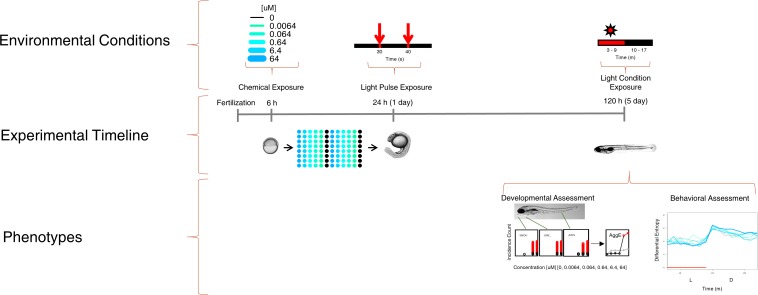
Experimental Design. Experimental timeline for chemical exposures at concentrations {0uM, 0.0064uM, 0.064uM, 0.64uM, 6.4uM, 64uM} added at 6hpf, with n = 32 embryos per concentration. At 24hpf, a nondestructive assay including two one-second light perturbations at 30s and 40s was performed (Details about this 24hpf behavioral assessment can be found at Reif et al. 2015). At 120hpf, behavior was measured under environmental conditions of 7 minutes continuous light exposure followed by an 8 minutes of dark. Behavioral and developmental (morphological) assessments were then recorded.

Fig 1 illustrates experimental perturbations (above the timeline), and associated analyses (below the timeline). Adult Tropical 5D zebrafish were housed at Sinnhuber Aquatic Research Laboratory at Oregon State University. All experiments were approved by the Institutional Animal Care and Use Committee (IACUC) of Oregon State University. Each tank was kept at 28°C on a 14h light/ 10h dark photoperiod. Group spawns of adult zebrafish were set up the night prior, and embryos were collected and staged [13]. Embryo chorions were enzymatically removed using pronase (90 μL of 25.3 U/μl; Roche, Indianapolis, In, USA) at 4 hours post fertilization (hpf) using a custom automated dechorionator and protocol described in Mandrell *et al*. [14]. Six hpf dechorinated embryos were placed individually into the wells of two 96-well plates per chemical using the automated embryo placement systems (AEPS) [14]. Chemicals were added to final well concentrations of 0, 0.0064, 0.064, 0.64, 6.4, and 64uM, with 0.64% DMSO included as the vehicle. Thus, there are 32 embryos per chemical per concentration. The layout of each concentration within a plate is shown in Fig 1. At 24hpf, an embryonic photomotor response (EPR) test was implemented [8]. After EPR, all exposed plates were wrapped with alumnium foil to prevent photodegradation, kept in a 28°C incubator, and statically exposed until 120hpf.

At 120hpf, zebrafish larvae movement was recorded in Viewpoint Zebrabox (Viewpoint Life Sciences, Lyon, France) during a 7-minute period of light followed by an 8-minute period of dark, then evaluated for 18 distinct morphological endpoints. The 18 morphological endpoints recorded for developmental assessment were Mortality (MORT), Yolk sac edema (YSE), Body axis (AXIS), Eye defect (EYE), Snout (SNOU), Jaw (JAW), Otic vesicle (OTIC), Pericardial edema (PE), Brain (BRAI), Somite (SOMI), Pectoral fin (PFIN), Caudal fin (CFIN), Pigmentation (PIG), Circulation (CIRC), Truncated body (TRUN), Swim bladder (SWIM), Notochord & Bent tail (NC), and Touch response (TR). Each morphological endpoint was recorded as a binary presence/absence according to the protocol detailed in [12]. All data were recorded by the Zebrafish Acquisition and Analysis Program (ZAAP) [12]. The current manuscript primarily focuses on the 120hpf behavioral assessment (see right-most ‘Light Condition Exposure’ portion of Fig 1).

### Statistical framework

**Fig 2:**
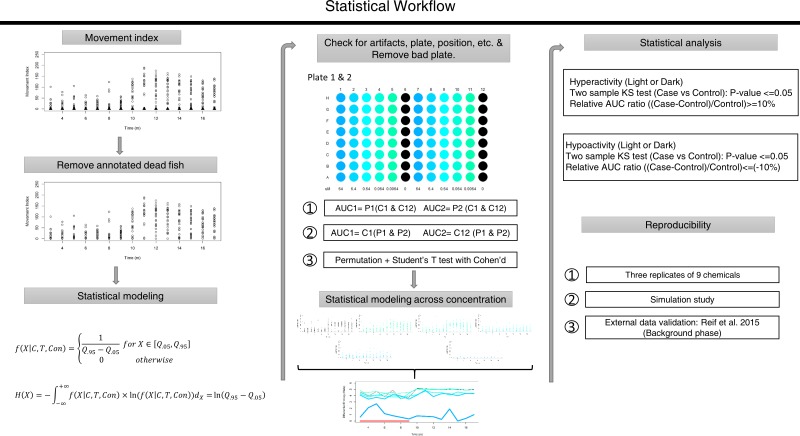
Statistical Workflow. Step 1: Visualize movement index; Step 2: Remove annotated dead fish for every concentration of a chemical; Step 3: Propose a statistical modeling method; Step 4: Check for artifacts, such as technical issues, global plate and position effect, and remove any bad plates; P1: Plate 1; P2: Plate 2; C1: Column 1; C12: Column 12; Step 5: Apply statistical modeling method to provide dose-response patterns for analysis; Step 6: Statistical analysis pipeline; Step 7: Assess reproducibility of our computational framework.

Data processing and all statistical analysis were implemented using custom R software [15]. The statistical pipeline is summarized in Fig 2. The movement index (total distance moved per unit time) was plotted for each minute across the experimental time period. Next, the annotated dead fish were removed prior to statistical modeling and analysis. For some chemicals, all embryos were dead at higher concentration(s) during exposure. These scenarios were reported as missing data, and were not processed in the next step. Using the remaining data, we applied our novel statistical approach.

We treated the movement as multiple uniform distributions across the experimental time period, where *X*, *C*, *T*, and *Con* represent individual movement, chemical, time point, and concentration respectively. At each time point, instead of using the maximum and minimum movement as the parameters for uniform distribution, we chose the 95% and 5% quartile movement, written as *Q*_.95_ and *Q*_.05_ such that we could reduce the bias caused by potential outliers at both directions. Therefore, the probability density function of our defined uniform distribution can be written as:

$$f(X|C,T,Con)=\left\{ \begin{array}{l} \frac{1}{Q_{.95}-Q_{.05}}\;for\;X\in [Q_{.95},Q_{.05}]\\ \;0\;otherwise \end{array}\right.$$

Our statistical modeling method is called differential entropy, which allows us to measure the average surprisal of continuous probability distributions [16]. The differential entropy *h*(*X*) in *nats* unit of our defined uniform distribution can be written as:

$$h(X|C,T,Con)=-\int_{-\infty }^{+\infty }f(X|C,T,Con)\times \ln(f(X|C,T,Con))d_{X}=\ln\left(Q_{.95}-Q_{.05}\right)$$

Taken together, the distribution of transformed movement is determined by connecting the differential entropy across time.

Next, we used unexposed controls to check for global artifacts detectable as plate effects. There are 3 aspects to this step: 1) Check for bad plates by extracting the area under the curve (AUC) ratio of two plates within a chemical, written as AUC1/AUC2 as shown in Fig 2. We calculate AUC as the following: let *T* = {*t*_1_,…,*t*_15_} represent time with unit *m*, and *H* = {*h*_1_,…,*h*_15_} represent the set of differential entropy at each time point with unit *nats*, thus the AUC is $\frac{h_{1}+2*(h_{2}+\cdots +h_{14})+h_{15}}{2}$ with unit nats*m. The ratios are expected to be close to 1, which indicates a fair experimental design. Suspicious large or small ratios were inspected individually before a decision was made to remove bad plates. 2) In our plate design, controls were placed in two different columns of each plate (one inner plus one outer). So we separated the controls of each chemical by position and followed the same procedures of checking for aberrant plates. 3) Since our plate number was assigned artificially during the experiment, we carried out a permutation test to randomly shuffle the plates of each chemical and looked for global plate and position effects. In each permutation, for each chemical, we generated a random number between 0 and 1. If the number was greater than 0.5, we accepted the ratio as AUC1/AUC2, otherwise the ratio was defined as AUC2/AUC1. We did this for both plate and position, and calculated Students’ t-test statistics using the null hypothesis that the ratio = 1 and Cohen’s D value as effect size of each permutation (1,000 rounds).

Next, we plotted the differential entropies of each chemical across time and concentrations to visualize the chemical-associated behavioral pattern. And the whole experimental period was separated into two intervals for statistical analysis: Light (minutes 3–9) and Dark (minutes 10–17).

Our statistical analysis framework for determining significant chemical-associated behavioral changes is modified from Reif et al. [8]. We found that the mean relative AUC ratio and actual AUC ratio of each interval follow a perfect linear relationship using our modeling method, thus in this study, we implemented the actual AUC ratio as a co-measurement to reflect the observed variability of the 120hpf behavioral data. Our statistical significance thresholds are presented in Fig 2.

Finally, we assessed the reproducibility of our statistical modeling and analysis framework using the independent replicates of 9 chemicals, a simulation study, and external data validation using the background (pre-stimulation) phase of the 24hpf behavioral assay described in [8].

### Data integration

Integrated analysis across morphological endpoints and meta-analysis looking at *in vivo* mammalian data was used to validate results and explore mechanistic hypotheses related to chemical bioactivity. For 120hpf morphological endpoints, the comprehensive developmental Aggregate Entropy (AggE) score for each chemical was used, as described in Zhang et al. [17]. For meta-analysis, the Toxicity Reference Database (ToxRefDB), which contains up-to-date in vivo animal toxicity studies of over 800 chemicals, was downloaded from US EPA [18]. ToxRefDB contains a lot of missing data, thus our decision of making a significant call is controlled by: 1) the lower bound of the 95% confidence interval of relative risk is greater than 1; 2) the p value of Fisher’s Enrichment Test is less than 0.05.

## Results

### Overview of all controls

**Fig 3:**
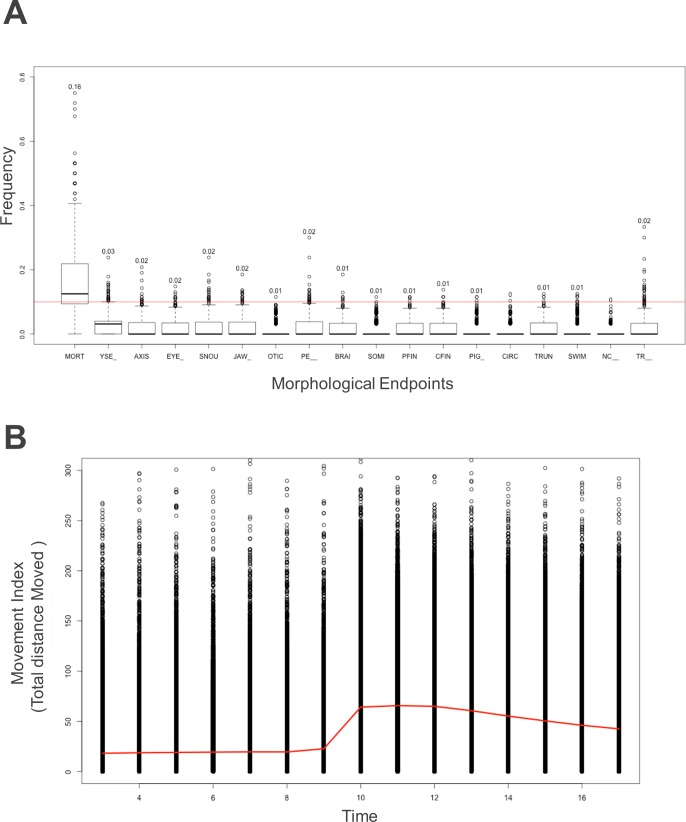
Overview of Controls. **A: Morphological overview of controls.** Incidence rate assessment (Y axis) for all chemicals by endpoint (X axis). For mortality, rate was calculated with a sample size of 32. For other endpoints, rate was calculated conditionally on alive zebrafish larvae. The red line was drawn at 10% for visualization. **B: Movement index overview:** Plot of healthy (i.e. no annotated morphological endpoints) zebrafish larvae in control wells for all plates. Red line was drawn by connecting the mean of each experimental time point. Y axis: Movement Index; X axis: Time (minutes).

Each morphological endpoint across all 1,060 control groups is shown in Fig 3A. For mortality, the observed frequency is $\frac{\sum Incidences}{32}$; for any other endpoint, the frequency was calculated by $\frac{\sum Incidences}{32-Mortality}$. As in Fig 3A, mortality is the only endpoint observed at an appreciable frequency among controls. Therefore, after removing annotated dead fish, the behavioral assessment of the controls should provide a robust baseline comparison. Because of the nature of this behavioral test, we should expect basal movement during the light phase and an excitement during the dark phase. To visualize this nature, we aggregated all healthy controls (i.e. those lacking any annotated morphological endpoint), and found that there was a high level of variation among the behavioral profiles (Fig 3B). Additionally, the difference between light and dark phases is likely underestimated prior to application of our new approach because of a large number of nonresponding individuals.

### Handling observed variation

**Fig 4:**
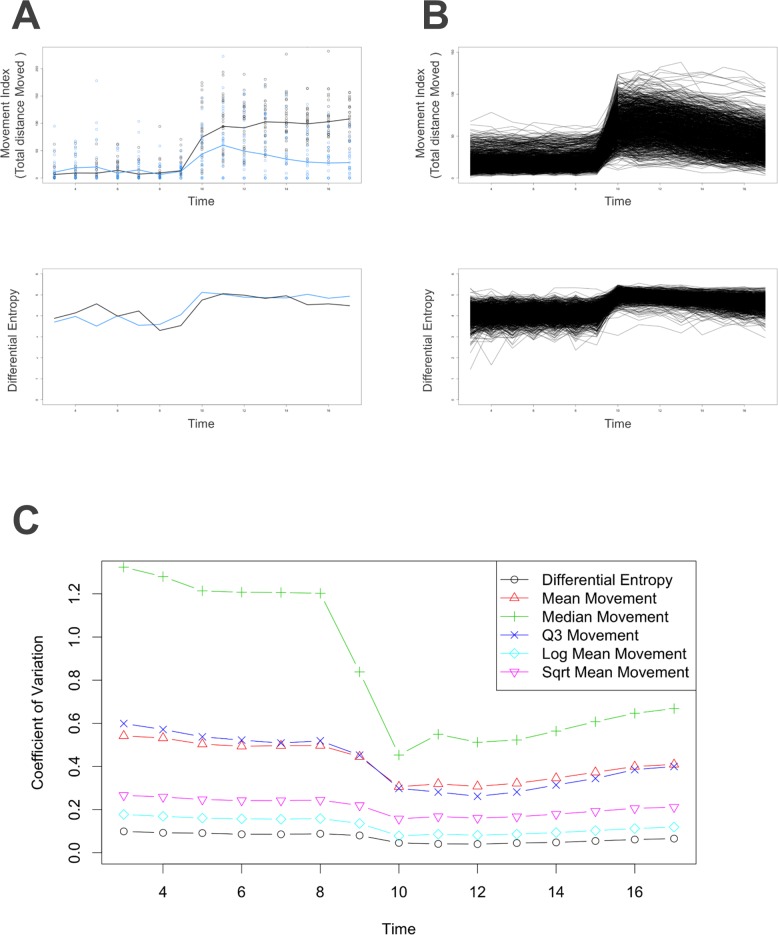
Performance of Our Novel Statistical Modeling Method. **A:** Example controls (having similar survival rates) illustrate the transformation. These two separate controls were plotted by different colors. Blue: TX000769 (Propoxycarbazone-sodium); Black: TX000900 (Methamidophos). Top: Movement index of each time point, and the line was drawn by connecting the mean movement indexes. Y axis: Movement index; X axis: Time. Bottom: Lines were drawn using our method, which connects the differential entropy of each time point. Y axis: Differential entropy (Nats); X axis: Time. **B:** All 1,060 control groups were plotted. Top: Each line represents a chemical. Line was drawn by connecting mean movement indexes. Y axis: Movement index; X axis: Time. Bottom: Each line represents a chemical. Line was drawn by connecting differential entropy across time. Y axis: Differential entropy (Nats); X axis: Time. **C:** Coefficient variation of each time point using all control groups and various statistical modeling methods. Y axis: Coefficient of Variation; X axis: Time.

We applied the novel statistical approach outlined in *Methods* to appropriately handle the variation in the behavioral data, and avoid bias caused by the skewed distribution of the actual movement at each time point. We illustrated this point in Fig 4, where movement traces of vehicle controls from two example chemicals (nominally called control 1 and 2), and provided the transition from traditional modeling (mean movement) to our statistical modeling method (Fig 4A, top). These two groups behaved similarly in the light phase; however, we observed an apparently stark difference during the dark phase. After checking individual movement at each time point, we found that these two groups shared a similar movement range. By implementing our statistical modeling method, these two control groups became consistent (Fig 4A, bottom). We plotted all 1,060 control groups and found that our statistical modeling method significantly improved the consistency among the controls (Fig 4B).

Our method provided a consistent evaluation over the controls and significantly reduced the coefficient of variation compared to various traditional modeling methods, including mean movement, simple logarithm transformed mean movement, the third quartile movement, median movement, and the square root of mean movement (Fig 4B and 4C). To further validate the consistency of our method, we provided a histogram with empirical density of the AUC ratios using the methods described above (S1 Fig). Each ratio was determined by choosing two control groups and calculated their AUC as AUC1 and AUC2. The ratio is AUC1/AUC2. We have a total of 561,270, which is (10602), ratios for each method.

### Check for artifacts

**Fig 5:**
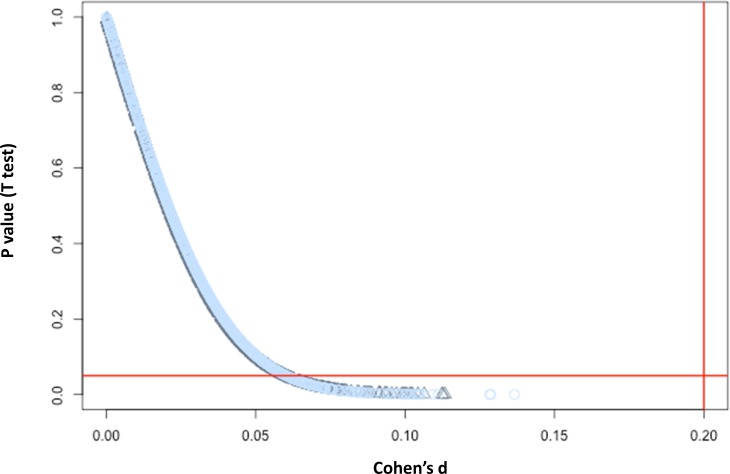
Statistics for checking artifacts. P value and Cohen’s d from each permutation was plotted (Black represents plate; Blue represents position). Y axis: Student’s t test p value; X axis: Cohen’s d. Horizontal red line was drawn at a significance level of 0.05. Vertical red line was drawn at 0.2 to represent the general rule of thumb of effect size.

We first used the pre-labeled plate number to construct the behavioral AUC ratio, AUC1/AUC2 as described in the *Methods* section, to detect for unusual plates using the control group. Our results showed 14 suspicious ratios, which are either infinity or zero. We selected these chemicals and inspected them individually to detect the issue. We found that in these 14 chemicals, one plate shows many identical movements across time not only for the controls but any other concentration. Checking against ZAAP records and annotation, we determined these to be experimental artifacts and removed these 14 plates prior to the next step, meaning that each of these 14 chemicals had half of the typical sample size for every concentration for the final analysis. We next investigated whether there was a similar issue for the remaining locations and found that, after removal of the offending plates, there were no extremely large or small ratios observed. Furthermore, our permutation study results (Fig 5) indicated that these 14 plates happened at random, and there were neither global plate nor positional effects.

### Reproducibility of our statistical pipeline

Evaluation of the reproducibility of our statistical pipeline includes three aspects: three independent replicates of each of the 9 chemicals, simulation study, and external data validation. For each chemical-concentration-interval, if the activity calls are the same among all three replicates, then the reliability is recorded as 1, otherwise it is 0. We achieved an overall 83% reliability, indicating good consistency of our method. Next, we did a simulation study for all chemicals and compared the significant activity call using the original data. We performed 500 simulations per chemical-concentration-interval for all 1,060 chemicals, and calculated the concordance between simulated activity calls to those generated by the original data. We found that the lowest mean concordance is over 97%, a strong indicator of the robustness of our pipeline. Finally, we used the previous published data from the photomotor response assay (PMR) to validate our method. Only the background phase, prior to light perturbations, was used for further evaluation since the environmental nature is similar to the light phase of our experiment. If a chemical was active using our method in at least one concentration, it was recorded as 1, otherwise 0. The same activity call was made from the Reif et al. [8] behavioral profiles. The concordance between these two pipelines is 95%. The main reason for the disagreement is that instead of using the mean AUC, our new method implemented the exact AUC to reflect the actual behavioral changes. Unlike the 120hpf behavioral data, the PMR assessed movement by each second, thus, the movements between two consecutive seconds can be very different.

### Summary of statistical analysis results

**Fig 6:**
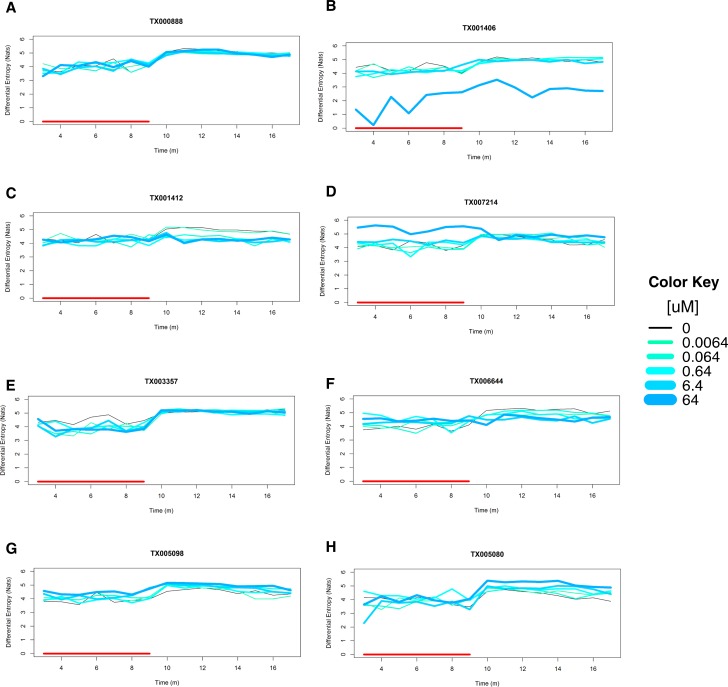
Behavioral response patterns. Differential entropy of each concentration was plotted across experimental time. Color key is shown in Fig 1. Y axis: Differential entropy (Nats); X axis: Time (m). Red segments represent light condition from 3m to 9m. **A: Inactive:** TX000888 (Terbacil) was inactive at all concentrations. **B: Hypoactivity (L) and Hypoactivity (D):** TX001406 (Cyclanilide) shows significant hypoactivities at 64uM for both light and dark intervals. **C: Inactive (L) and Hypoactivity (D):** TX001412 (Fipronil) is inactive at light interval and shows significant hypoactivity at dark interval at 0.064uM, 0.64uM, 6.4uM, and 64uM. **D: Hyperactivity (L) and Inactive (D):** TX007214 (Dieldrin) shows significant hyperactivity at light interval but it is inactive at dark at 64uM. **E: Hypoactivity (L) and Inactive (D):** TX003357 (44’-Oxydianiline) shows significant hypoactivity at light interval and inactive pattern at dark interval at 0.064uM, 6.4uM, and 64uM. **F: Hyperactivity (L) and Hypoactivity (D):** TX006644 (Haloperidol) shows significant hyperactivity at light interval and significant hypoactivity at dark interval at both 6.4uM and 64uM. In addition, at 0.64uM, it shows significant hyperactivity at light interval. **G: Hyperactivity (L) and Hyperactivity (D):** TX005098 (4-Pentylaniline) shows significant hyperactivity at 64uM for both light and dark conditions. **H: Inactive (L) and Hyperactivity (D):** TX005080 (44’4”-Ethane-111-triyltriphenol) is inactive at light and shows significant hyperactivity at dark at 6.4uM and 64uM.

There are 356 chemicals that showed significant hypoactivity or hyperactivity in at least one interval. Of these significant chemicals, 193 chemicals were only found significant in the light interval, 83 chemicals were only significant in the dark interval, and 80 chemicals were significant in both intervals. All behavioral profiles of these 1,060 chemicals can be found in the S1 File. Exploring patterns of chemical-elicited hypoactivity and hyperactivity across intervals may suggest different key biological events or pathway perturbations, so we summarized those 356 significant behavioral profiles through various behavioral effect patterns of each concentration (Table 1). The example of each behavioral pattern is shown in Fig 6A–6F. The global differential entropies of the control groups are 4.10 for the light phase and 4.82 for the dark phase. The 95% confidence intervals are (3.38, 4.81) and (4.29, 5.35) for light and dark respectively. An example of nonsignificant behavioral pattern is shown in Fig 6A. In general, we observed that of these chemicals that were significant in both intervals, hypoactivity (Light)-hypoactivity (Dark) (Fig 6B) is the most common pattern, and the number of chemicals goes up as the concentration increases. The same trend was observed in the inactive (Light)-hypoactivity (Dark) (Fig 6C) behavioral pattern. About 40 chemicals were found at hyperactivity (Light)-inactive (Dark) (Fig 6D) pattern, and 20 chemicals at hypoactivity (Light)-inactive (Dark) (Fig 6E) in every concentration. Behavioral effect patterns, such as hyperactivity (Light)-hypoactivity (Dark) (Fig 6F), hyperactivity (Light)-hyperactivity (Dark) (Fig 6G), and inactive (Light)-hyperactivity (Dark) (Fig 6H), are very rare. No chemicals were observed at hypoactivity (Light)-hyperactivity (Dark).

**Table 1:** Summary of statistically significant behavioral profiles.

Behavioral Effect Pattern	0.0064uM	0.064uM	0.64uM	6.4uM	64uM
Light (Hypoactivity)	1	1	2	12	55
Dark (Hypoactivity)					
Light (Hypoactivity)	0	0	0	0	0
Dark (Hyperactivity)					
Light (Hyperactivity)	0	1	1	3	4
Dark (Hypoactivity)					
Light (Hyperactivity)	0	0	0	0	2
Dark (Hyperactivity)					
Light (Hypoactivity)	14	18	22	18	26
Dark (Inactive)					
Light (Inactive)	4	4	11	23	62
Dark (Hypoactivity)					
Light (Hyperactivity)	30	37	47	39	36
Dark (Inactive)					
Light (Inactive)	5	8	4	11	10
Dark (Hyperactivity)					

### Data integration

**Fig 7:**
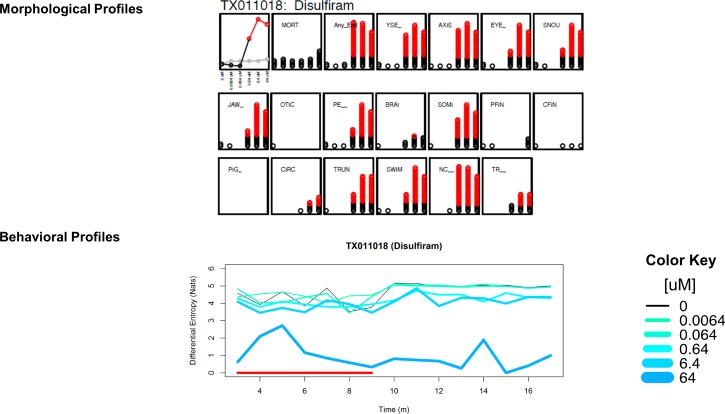
Relationship between morphological profiles and behavioral profiles. Disulfiram significantly affected 13 endpoints starting at 0.64 uM. Disulfiram also caused significant hypoactivity in both intervals with a lowest effect level of 0.64 uM. For morphological profiles, the panels represent (from top left) Aggregate Entropy, mortality, summation of any endpoint, then each of the specific endpoints (see ‘Methods‘). The X axes show concentration (0uM, 0.0064uM, 0.064uM, 0.64uM, 6.4uM, 64uM from left to right). The Y axes show Aggregate Entropy for the first panel, then incidence counts for all other panels. Red indicates statistical significance for each measure (p < 0.05).

We next investigated whether there was a connection between chemical-associated activity call and developmental malformations. Of those chemicals that were significant in our 120hpf behavioral assay, for every significant concentration, we found the corresponding AggE from Zhang et al. [17]. For non-significant chemicals, we provided the highest AggE from Zhang et al. We performed a two sample Student’s t-test and concluded that chemicals that were significantly affecting behavior tended to have higher AggE. An example of this connection can be found in Fig 7.

Taking advantage of this complex and systematic behavioral assessment in zebrafish, we integrated this assay with ToxRefDB toxicity endpoints. We defined our significance vector as follows: if a chemical is significant in at least one interval, it is recorded as 1, otherwise 0. Our statistics were calculated based on a binary table between our assay and the significance matrix of ToxRefDB. Those ToxRefDB endpoints that meet the criteria are shown in S1 Table. We found that all significantly enriched ToxRefDB endpoints are systemic carcinogens related endpoints except for a few in developmental reproductive studies.

## Discussion

In the present study, we provided a large-scale systematic behavioral assessment using zebrafish to characterize bioactivity of a diverse ToxCast Phase-I and Phase-II libraries. This multiple concentration-response design allowed us to fully capture the behavioral changes and provided increased power for detecting significant chemical-elicited activities. Behavioral assessments are complex phenotypes that are difficult to measure and characterize even without chemical perturbants. Therefore, traditional statistical methods may not be appropriate due to limiting assumptions and poor performance in the face of high variability.

Faced with these challenges, we developed a novel computational framework and evaluated its performance on a variety of fronts. Even in healthy controls, behavioral endpoints may show dramatic variation, which makes appropriate handling of that variation all the more important for analysis of chemical treated embryos. We applied permutation approaches to the quality control step and checked for technique artifacts and removed bad plates. After our statistical treatment, the average coefficient of variation of the controls across time was significantly reduced compared to many other modeling techniques. Our method also performed well on data having lower sample size (demonstrated by its consistent performance on the chemicals for which a problematic experimental plate was deleted). Across chemicals compared using controls, our modeling method showed great consistency over traditional mean movement modeling (S1 Fig). To this end, we encourage the application of our method to other types of behavioral data, and small-scale studies to further validate its performance.

Data integration provided insights to the biological relevance of this behavioral assay. There is a connection between altered behavioral phenotypes and developmental malformations. Integrating these two measurements could increase the reliability of hazardous assessments of these chemicals. Moreover, by mapping zebrafish behavioral profiles to ToxRefDB, we were able to identity many significant systematic carcinogenicity endpoints, suggesting zebrafish are a good alternative model for toxicological research. We also found that many chemical analogs shared similar bioactivity, suggesting that these behavioral data could be used to build quantitative structure-activity relationship (QSAR) models that predict responses to as-yet untested compounds. For example, the well-known endocrine disruptors kepone and the thyroid hormone 3,5,3’-Triiodothyronine shared similar profiles following application of our analysis framework. These two chemicals were both found active in US EPA Endocrine Disruptor Screening Program for the 21^st^ Century (EDSP21) (http://actor.epa.gov/edsp21/). Methyleugenol shared similar behavioral effects yet was inactive in all of the *in vitro* assay tested at EDSP21. Therefore, appropriate behavioral profiling using zebrafish can identify chemicals with similar biological processes.

**Fig 8:**
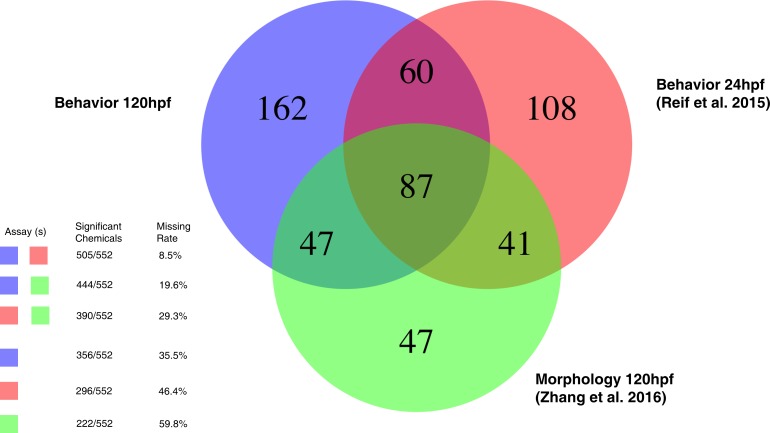
Summary of results by the whole experimental system. This Venn diagram provides the summary of the total number of significant chemicals detected by each assay. It also provides statistics regarding the benefits of including all assays. Missing rate: the number of chemicals that would have been missed using a subset of these assays.

An active and important area of toxicological research is to develop risk-based prioritization methods for chemicals that need further testing. This zebrafish behavioral assay can be implemented together with existing hazard data for risk assessment of environmental chemicals. Integrating multiple data streams increases the detection power for hazardous chemicals, especially for endpoints that are only assayable in organismal systems. As in Fig 1, the whole experimental system contains three assays: a 24hpf behavioral assay [8], a 120hpf morphological assay [12, 17], and a 120 behavioral assay. A summary of the number of significant chemicals detected per assay collection is shown in Fig 8. Across all assays, there were 552 chemicals eliciting a statistically significant response. It is apparent from Fig 8 that excluding an assay will result in reduced detection of chemicals with potential hazard across developmental time points and effect categories. In contrast, integration across the entire experimental system can provide useful information for risk assessment of environmental chemicals by providing multiple measurements across a developmental timeline and varying environmental conditions.

In conclusion, HTS behavioral studies using zebrafish provides systematic data that can be used for integrated analysis. Behavioral phenotypes are complex and may appear noisy when not appropriately analyzed. Our new statistical modeling method and pipeline help make more robust decisions regarding chemical-associated behavioral effects by reducing coefficients of variation, increase consistency among different chemicals, and quantifying reproducibility. Moreover, appropriate behavioral profiling and data integration adds meaningful context that can inform mechanistic hypotheses.

## Supporting Information

S1 Fig Comparison of various statistical modeling methods by plotting the density of the AUC ratios of any pair of control groups. Ratio was censored at 3 to preserve visualization of the variation. Y axis: Density; X axis: AUC ratio.
(TIF)

S1 File Behavioral profiles of all chemicals. (CSV)

S1 Table Significantly enriched ToxRefDB endpoints. (PDF)
