# Supplementary figures and images for: A New Statistical Approach to Characterize Chemical-Elicited Behavioral Effects in High-Throughput Studies Using Zebrafish
Source: PLoS One. 2017 Jan 18;12(1):e0169408. doi: 10.1371/journal.pone.0169408 (PMC5242475; doi:10.1371/journal.pone.0169408)

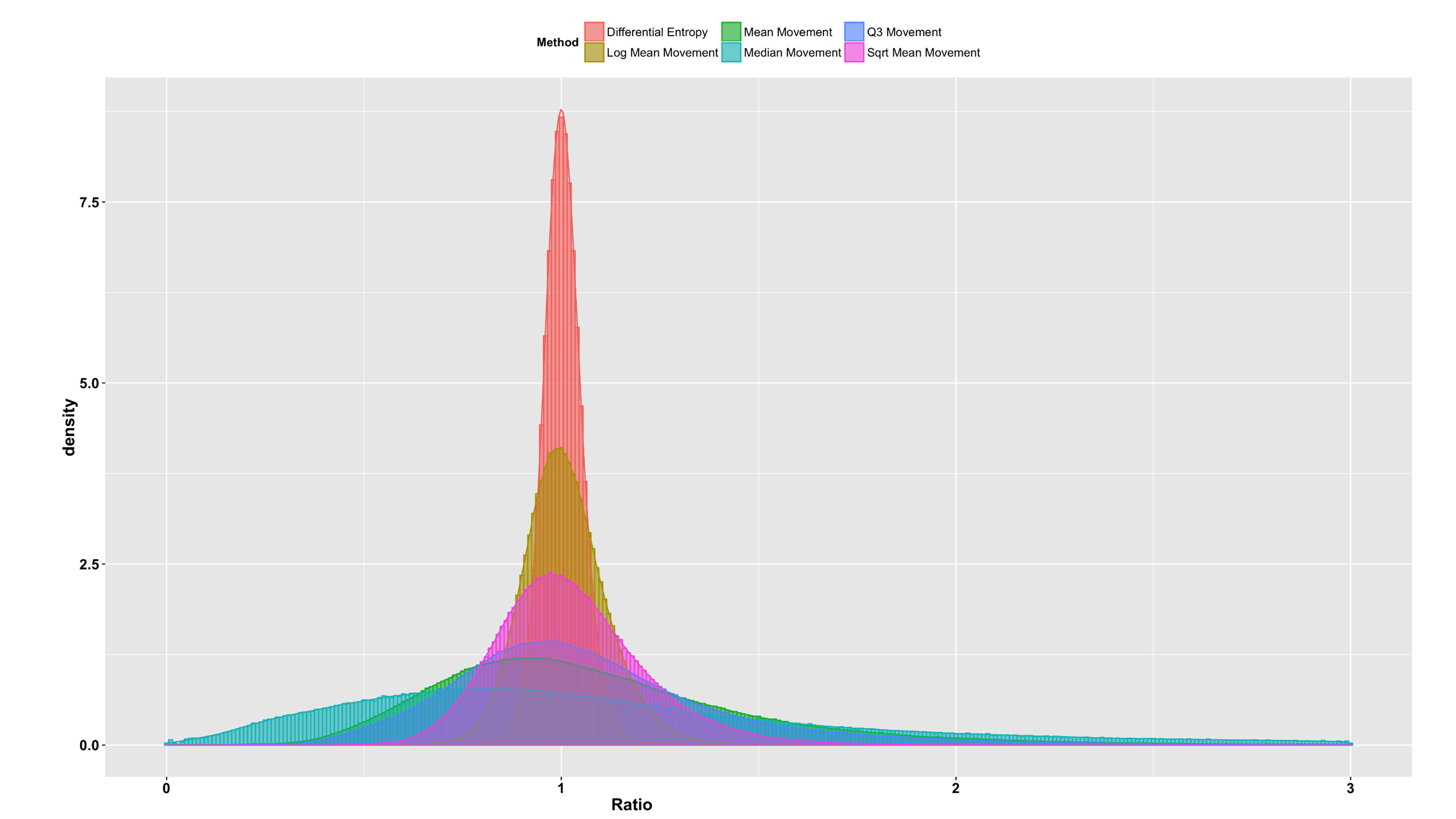

Supplement: S1 Fig — Ratio was censored at 3 to preserve visualization of the variation. Y axis: Density; X axis: AUC ratio. (TIF) [file pone.0169408.s001.TIF]
