# Supplementary material for: A New Statistical Approach to Characterize Chemical-Elicited Behavioral Effects in High-Throughput Studies Using Zebrafish
Source: PLoS One. 2017 Jan 18;12(1):e0169408. doi: 10.1371/journal.pone.0169408 (PMC5242475; doi:10.1371/journal.pone.0169408)
Supplement: S1 Table — (PDF) [file pone.0169408.s003.pdf]

Endpoint	Concordance	RelativeRisk	FisherEnrichment
SUB_dog_SystemicCarcinogenic_adult_OrganWeight_Nervous	0.612	8.105	0.042
SUB_mouse_SystemicCarcinogenic_adult_PathologyProliferative_PrimaryDigestive	0.702	6.92	0.007
SUB_mouse_SystemicCarcinogenic_adult_PathologyProliferative_PrimaryDigestive_Stomach	0.695	5.932	0.017
SUB_rat_SystemicCarcinogenic_adult_AccessoryDigestive_Pancreas	0.627	4.151	0.027
CHR_rat_DevelopmentalReproductive_adult_PathologyNonProliferative_ReproductiveMale_Epididymis	0.662	4.119	0.015
CHR_mouse_SystemicCarcinogenic_adult_PathologyGross_PrimaryDigestive_Stomach	0.644	3.979	0.042
CHR_rat_SystemicCarcinogenic_adult_PathologyNonProliferative_Lymphatic_LymphNode	0.665	3.661	0.009
CHR_rat_SystemicCarcinogenic_adult_PathologyNonProliferative_AccessoryDigestive_Pancreas	0.665	3.661	0.009
CHR_mouse_DevelopmentalReproductive_adult_PathologyGross_ReproductiveMale	0.644	3.411	0.036
CHR_rat_SystemicCarcinogenic_adult_PathologyProliferative_PrimaryDigestive_Stomach	0.659	3.295	0.041
CHR_rat_DevelopmentalReproductive_adult_PathologyNonProliferative_ReproductiveMale_Prostate	0.659	3.295	0.041
CHR_rat_SystemicCarcinogenic_adult_Lymphatic_LymphNode	0.668	3.112	0.003
CHR_rat_SystemicCarcinogenic_adult_OrganWeight_Respiratory	0.662	2.975	0.016
SUB_mouse_SystemicCarcinogenic_adult_PrimaryDigestive_Stomach	0.687	2.966	0.038
MGR_rat_DevelopmentalReproductive_pregnancy_ReproductiveMating_ReproductivePerformance_Gestationalinterval	0.626	2.654	0.048
SUB_dog_SystemicCarcinogenic_adult_OrganWeight_Urinary	0.619	2.509	0.048
CHR_mouse_SystemicCarcinogenic_adult_PathologyNonProliferative_Endocrine_AdrenalGland	0.644	2.325	0.032
MGR_rat_DevelopmentalReproductive_pregnancy_ReproductiveMating_OffspringSurvivalEarly_Livebirthindex	0.634	2.271	0.01
SUB_rat_SystemicCarcinogenic_adult_OrganWeight_Cardiovascular	0.638	2.036	0.003
CHR_rat_DevelopmentalReproductive_adult_ReproductiveFemale_Uterus	0.65	1.927	0.032
CHR_mouse_SystemicCarcinogenic_adult_PathologyNonProliferative_Endocrine	0.637	1.885	0.035
SUB_rat_SystemicCarcinogenic_adult_Cardiovascular_Heart	0.62	1.707	0.014
CHR_rat_DevelopmentalReproductive_adult_PathologyNonProliferative	0.638	1.664	0.028
CHR_rat_SystemicCarcinogenic_adult_Cardiovascular_Heart	0.638	1.659	0.037
CHR_rat_SystemicCarcinogenic_adult_OtherSystemic_InLifeObservations_FoodConsumption	0.635	1.638	0.025
CHR_rat_SystemicCarcinogenic_adult_Nervous_Brain	0.635	1.609	0.04
CHR_rat_SystemicCarcinogenic_adult_Respiratory_Lung	0.629	1.556	0.038
MGR_rat_DevelopmentalReproductive_pregnancy_ReproductiveMating_OffspringSurvivalEarly	0.608	1.548	0.02
CHR_mouse_SystemicCarcinogenic_adult_PathologyGross	0.607	1.471	0.029
SUB_rat_SystemicCarcinogenic_adult_OtherSystemic_InLifeObservations_ClinicalSigns	0.594	1.451	0.012
CHR_mouse_SystemicCarcinogenic_adult_PathologyNonProliferative_AccessoryDigestive_Liver	0.578	1.337	0.031
DEV_rat_SystemicCarcinogenic_adult_OtherSystemic_InLifeObservations_ClinicalSigns	0.557	1.319	0.003
CHR_mouse_SystemicCarcinogenic_adult_OrganWeight_AccessoryDigestive	0.571	1.312	0.042
CHR_rat_SystemicCarcinogenic_adult_OrganWeight_AccessoryDigestive	0.56	1.278	0.039
MGR_rat_SystemicCarcinogenic_juvenile_OtherSystemic_InLifeObservations_BodyWeight	0.506	1.194	0.006
MGR_rat_SystemicCarcinogenic_juvenile_OtherSystemic_InLifeObservations	0.498	1.174	0.012
CHR_rat_SystemicCarcinogenic_adult_PathologyNonProliferative	0.482	1.164	0.026
DEV_rat_SystemicCarcinogenic_adult_OtherSystemic_InLifeObservations	0.443	1.104	0.032
